# *In vitro* modeling of hepatocellular carcinoma molecular subtypes for anti-cancer drug assessment

**DOI:** 10.1038/emm.2017.164

**Published:** 2018-01-05

**Authors:** Hadassa Hirschfield, C Billie Bian, Takaaki Higashi, Shigeki Nakagawa, Tizita Z Zeleke, Venugopalan D Nair, Bryan C Fuchs, Yujin Hoshida

**Affiliations:** 1 Division of Liver Diseases, Department of Medicine, Liver Cancer Program, Tisch Cancer Institute, Graduate School of Biomedical Sciences, Icahn School of Medicine at Mount Sinai, New York, NY, USA; 2 Department of Gastroenterological Surgery, Graduate School of Medical Science, Kumamoto University, Kumamoto, Japan; 3 Department of Neurology, Icahn School of Medicine at Mount Sinai, New York, NY, USA; 4 Division of Surgical Oncology, Massachusetts General Hospital Cancer Center, Harvard Medical School, Boston, MA, USA

## Abstract

Tractable experimental model that accounts for inter-tumor molecular heterogeneity is a key element of anti-cancer drug development. Hepatocellular carcinoma is known to exhibit highly heterogeneous molecular aberrations across the tumors, including somatic genetic and epigenetic alterations. Previous studies showed that molecular tumor subtypes determined by transcriptome, as a comprehensive functional readout, are reproducibly observed across global patient populations irrespective of geographic and etiological variations. Here we demonstrate that transcriptomic hepatocellular carcinoma subtypes, S1 and S2, determined by our previous transcriptome meta-analysis of multiple clinical hepatocellular carcinoma cohorts, are presented in a panel of hepatoma cell lines widely used by the research community. Interestingly, cell line that resembles gene expression pattern of S3 subtype, representing less aggressive tumors, was not identified in the panel. MYC pathway-activated S2-like cell lines showed higher sensitivity to a small molecule BET bromodomain inhibitor, (+)-JQ1, which has anti-MYC activity. These results support the use of hepatoma cell lines as models to evaluate molecular subtype-specific drug response, which is expected to lead to development of tailored, precision care of the patients with hepatocellular carcinoma.

## Introduction

Experimental models are key components of anti-cancer drug development and an urgent unmet need for hepatocellular carcinoma (HCC), which still lacks substantially effective medical therapy.^1^ Extensive genomic analysis of HCC tumors has clarified significant inter- and intra-tumor molecular heterogeneity, which likely obscures therapeutic effect of molecular targeted agents. Potentially druggable somatic DNA mutations are generally rare (prevalence <5%), which makes patient enrollment in clinical trials more challenging.^2^ A recent study of patient-derived HCC cell lines demonstrated selective killing only in subclones that harbor the relatively rare somatic DNA aberrations such as *FGF19* gene amplification.^3^

Transcriptome is an alternative measure, which is assumed to be functional readouts of various types of molecular aberrations such as mutations and epigenetic changes. Our previous transcriptome meta-analysis revealed three subtypes, namely S1, S2 and S3, which are observed in larger fractions of patients/tumors (prevalence>20%) associated with specific molecular pathway dysregulation and patient prognosis.^1, 4, 5^ The subtypes are associated with clinical characteristics such as serum α-fetoprotein (AFP) levels, and also with histological intra-tumor heterogeneity, suggesting their clinical relevance.^5^ It is empirically known that some of the transcriptomic subtypes are present in human hepatoma cell lines, with which subtype-specific *in vitro* and *in vivo* anti-cancer drug response has been demonstrated.^6, 7, 8^ In parallel, clinical features associated with the subtypes such as AFP (a marker of the S2 subtype) have been recognized as possible predictive biomarkers of therapeutic response to molecular targeted agents as shown in recent clinical trials.^9^ These findings collectively suggest that hepatoma cell lines can be used as tractable and clinically relevant experimental models of the HCC subtypes for assessment of molecular targeted anti-cancer agents. In this study, we have systematically evaluated the HCC subtypes and their association with therapeutic response in a panel of frequently and widely used hepatoma cell lines.

## Materials and methods

### Transcriptomic subtyping of hepatoma cell lines

Pre-normalized genome-wide transcriptome and DNA mutation data sets of 25 hepatoma cell lines (Table 1) were downloaded from the Cancer Cell Line Encyclopedia (CCLE) website (https://portals.broadinstitute.org/ccle/home). Pre-normalized genome-wide transcriptome data set of 374 human HCC tissues was downloaded from The Cancer Genome Atlas (TCGA) website (https://gdc.cancer.gov). In the CCLE hepatoma data set, transcriptome profile of PLC/PRF/5 cell line duplicated in an alternative name (Alexander) was excluded. Hepatoblastoma-derived cell lines, Hep G2 and its derivative C3A,^10^ and undifferentiated hepatoma cell lines, HLE and HLF, were included in the analysis given their wide use in the HCC research community. Transcriptomic subtyping of the cell lines was performed based on the HCC subtype maker gene signatures by using the Nearest Template Prediction algorithm as previously reported.^4, 11^ Non-negative matrix factorization (NMF)-based consensus clustering was used to explore optimal number of classes in the global transcriptome data set after excluding less variable probes based on a coefficient of variation (CV) cutoff of 0.5.^12^

### Cell lines

SNU-182, SNU-387, SNU-475, SNU-449, Hep G2, Hep 3B2.1-7, Huh-7 and THLE-5B cells (American Type Culture Collection or Riken Bioresource, Japan) were grown in DMEM supplemented with 10% heat-inactivated fetal bovine serum, 100 U ml^−1^ penicillin (Gibco, Gaithersburg, MD, USA) and 100 μg ml^−1^ streptomycin (Gibco) at 37 °C in a 5% CO_2_ atmosphere.

### HCC subtyping with NanoString assay

Total RNA was extracted from the cell lines using RNeasy kit (Qiagen, Germantown, MD, USA), and 200 ng total RNA was subjected to the Elements HCC subtyping assay (NanoString, Seattle, WA, USA) as previously reported.^5^ Raw transcript count data were normalized by scaling with geometric mean of built-in normalization genes. HCC subtype determination was similarly performed by the Nearest Template Prediction algorithm.

### *In vitro* anti-HCC drug treatment

A total of 3000–5000 cells were plated in each well in 96-well plates according to growth rate of each cell line, and when 50% confluence was reached ~24 h later, treatment with (+)-JQ1 (Selleckchem, Houston, TX, USA) at varying concentrations was started and continued for 48 h in triplicates.

### Cell viability analysis

Cell viability was assessed using an MTS Cell Proliferation Assay (Promega, Madison, WI, USA). A volume of 22 μl of the MTS assay solution was added to each well that contained 110 μl of media. The plates were then incubated at 37 °C in a 5% CO_2_ atmosphere for 4 h. The absorbance was then read at 490 nm. Averaged absorbance from empty wells was subtracted from all wells as background signal, and relative cell viability was calculated as a percentage of the adjusted absorbance compared to respective DMSO-treated control wells.

### Statistical analysis

Proportion of categorical data was assessed by Fisher’s exact test. Two-tailed *P*-value <0.05 was regarded as statistically significant. All analyses were performed using R statistical language (www.r-project.org).

## Results

### Transcriptomic subtyping of hepatoma cell lines

**Figure 1:**
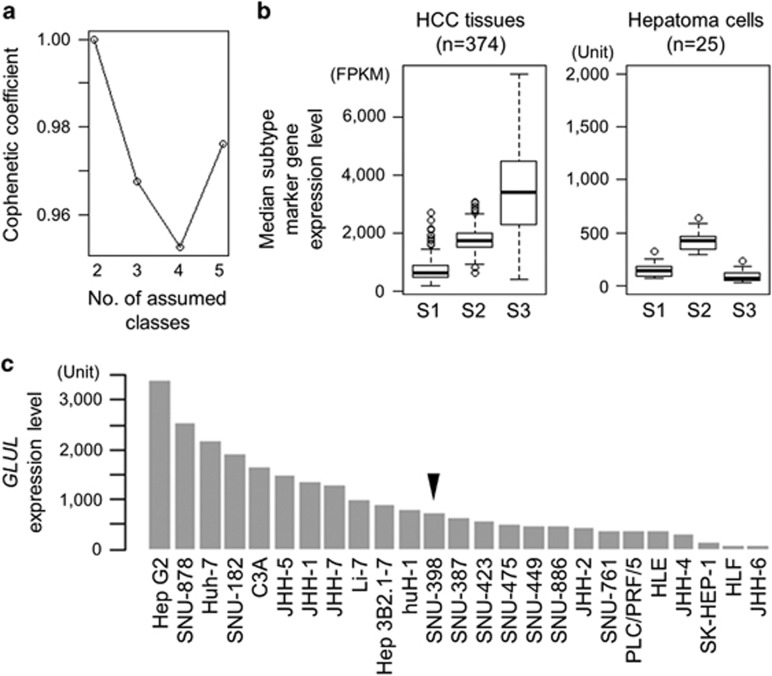
Transcriptomic hepatocellular carcinoma (HCC) subtypes and DNA mutations in 25 hepatoma cell lines. (**a**) Optimal number of unsupervised subclasses in the global transcriptome profiles of the 25 hepatoma cell lines from the Cancer Cell Line Encyclopedia (CCLE) data set was determined by cophenetic coefficient from non-negative matrix factorization (NMF) consensus clustering for each assumed number of subclasses, ranging from 2 to 5. (**b**) Median expression levels of the HCC subtype marker genes across the samples are plotted for each subtype in 374 clinical HCC tissues (left) and 25 hepatoma cell lines (right). Boxes represent 75th and 25th percentile, horizontal line is the median, and whiskers mark lowest and highest values. Outliers outside 1.5 × of inter-quartile range are shown as open circles. (**c**) *GLUL* expression level in the 25 hepatoma cell lines. Arrow head indicates a cell line with *CTNNB1* exon 3 mutation.

Our previous transcriptome meta-analysis involving 603 clinical HCC tissues revealed the following three subtypes: S1 subtype characterized by activation of stromal gene/pathway such as transforming growth factor β (TGFβ), S2 subtype with overexpression of stemness markers such as *EPCAM* and *AFP*, and S3 subtype enriched with more differentiated tumors and accumulation of *CTNNB1* exon 3 mutations associated with induction of liver-specific Wnt pathway target, *GLUL*.^13^ It has been reported that hepatoma cell lines are classified into two groups by unsupervised clustering of global transcriptome profiles, which is correlated with AFP expression status.^14, 15^ AFP-high and -low cell lines were empirically known to exhibit transcriptome profiles similar to S2 and S1 subtypes, respectively.^4^ Consistent with these observations, non-negative matrix factorization consensus clustering of the genome-wide transcriptome profiles of 25 hepatoma cell lines from the CCLE data set revealed that optimal classification is indeed achieved when two subclasses are assumed (Figure 1a). In the surgically resected clinical HCC tissues (*n*=374), median expression levels of each HCC subtype marker genes in each sample (S1: 238 genes, S2: 115 genes and S3: 266 genes) depicted relative abundance of each subtype marker gene expression in human HCC tissues (Figure 1b).

**Figure 2:**
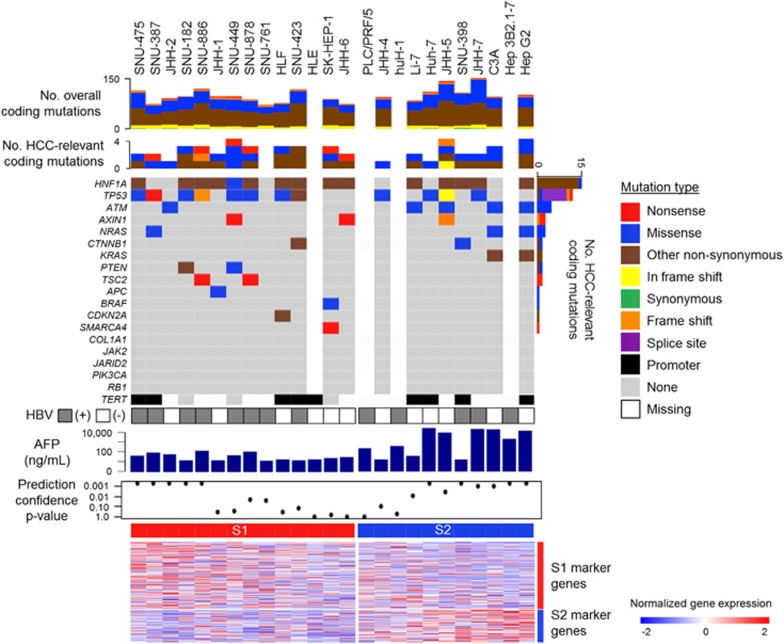
Transcriptomic hepatocellular carcinoma (HCC) subtypes and DNA mutations in 25 hepatoma cell lines. HCC subtypes were determined using genome-wide transcriptome data by the Nearest Template Prediction (NTP) algorithm as previously described.^4^ Expression pattern of the HCC subtype marker genes is shown in the heatmap.

In the hepatoma cell lines, the relative expression levels of the S1 and S2 subtype marker genes were maintained, whereas the S3 subtype marker genes were not expressed at the levels in tissues, suggesting that the hepatoma cell lines generally lack characteristic of the S3 subtype. In fact, the *CTNNB1* exon 3 mutations prevalent in the S3 subtype, was observed only in one cell lines (SNU-398) with relatively low expression of the target gene *GLUL* (Figures 1c and 2). These results collectively support that the hepatoma cell lines are classified into either the S1 or S2 subtype, and there is no cell line corresponding to the S3 subtype. In contrast, *TP53* gene mutations were more frequently observed (12 out of 22 cell lines with mutation data, 55%) as seen in clinical tissues, and *HIF1A* was the most frequently affected gene by mutations (68%). Prevalence of these mutations was similar between the HCC subtypes for *TP53* (*P*=0.67, Fisher’s exact test), *HIF1A* (*P*=0.38), *TERT* promoter (*P*=1.0) and rest of the genes recurrently affected in human HCC tissues (*P*=0.83).^2^

### Transcriptomic subtyping of hepatoma cells by an FDA-approved assay

**Figure 3:**
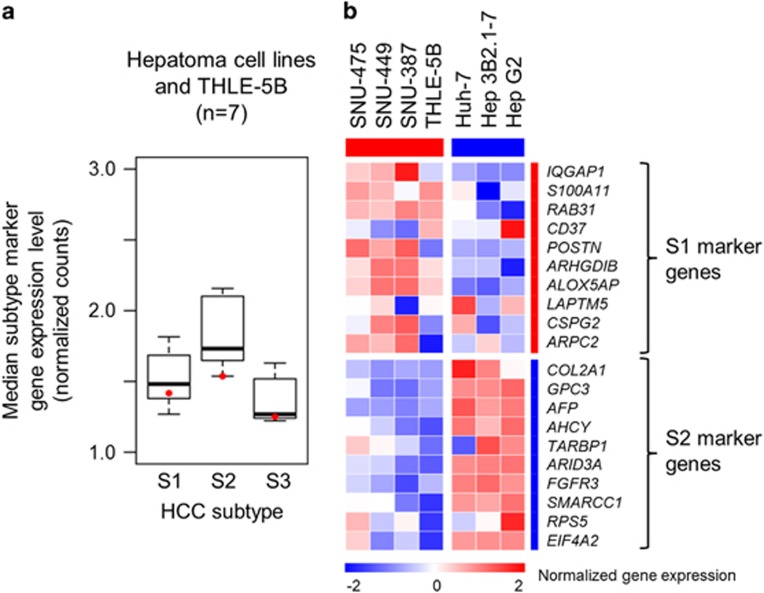
Transcriptomic hepatocellular carcinoma (HCC) subtypes determined by the NanoString assay. (**a**) Median expression levels of the HCC subtype marker genes are plotted for each subtype in seven cell lines. Red dots indicate expression in THLE-5B cells. Boxes represent 75th and 25th percentile, horizontal line is the median, and whiskers mark lowest and highest values. Outliers outside 1.5 × of inter-quartile range are shown as open circles. (**b**) Expression pattern of the HCC subtype marker genes in the seven cell lines is shown in the heatmap.

We have implemented a reduced version of the HCC subtyping gene signature (30-gene signature) in an FDA-approved diagnostic assay platform (NanoString) for clinical application in our previous study.^5^ The HCC subtype determination based on the genome-wide transcriptome data (Figure 2) was verified by using the NanoString assay for six representative hepatoma cell lines (SNU-465, SNU-449, SNU-387, Huh-7, Hep 3B and Hep G2) together with the non-tumorigenic SV40-immortalized hepatocyte line, THLE-5B.^16^ The relative expression of subtype S1 and S2 marker genes as well as low expression of S3 marker genes was verified in the hepatoma cells, and a similar pattern was observed in THLE-5B cells (Figure 3a). This result suggests that the lower expression of the S3 marker genes compared to HCC tissues is a common feature in established cell lines irrespective of origin, that is, malignant or non-malignant cells. The HCC subtype determination was also confirmed in the 30-gene HCC subtyping assay, supporting validity of the assay (Figure 3b).

### HCC subtypes are associated with response to molecular targeted agents

**Figure 4:**
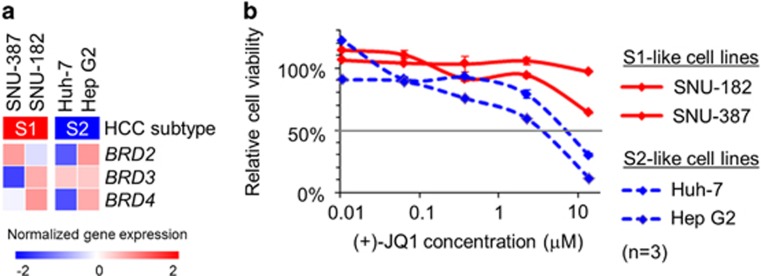
Transcriptomic hepatocellular carcinoma (HCC) subtype-dependent drug response in hepatoma cell lines. (**a**) Expression of *BRD2*, *BRD3* and *BRD4* genes, encoding targets of a BET bromodomain inhibitor, (+)-JQ1, in the S1-like (SNU-387 and SNU-182) and S2-like (Huh-7 and Hep G2) hepatoma cell lines in the CCLE data set. (**b**) Relative cell viability compared to DMSO-treated controls after 48 h treatment with (+)-JQ1 in a series of drug concentrations (range: 0.01–13.6 μM).

In our previous study, we could successfully demonstrate *in vitro* and *in vivo* subtype-specific drug response by targeting fibroblast growth factor receptor pathway, one of the molecular hallmarks specifically activated in the S2 subtype.^8^ We sought to extend this examination to another pathway implicated in HCC subtypes. MYC pathway is known to be activated in the S2 subtype.^4^ Direct targeting of this pathway has been technically challenging, but BET bromodomain inhibition by a small molecule, (+)-JQ1 has been show to elicit anti-MYC pathway activity.^17, 18^ To evaluate whether the subtype-dependent response to MYC pathway inhibition can be monitored in hepatoma cells, SNU-387 and SNU-182 (S1-like cells) as well as Huh-7 and Hep G2 (S2-like cells) were treated with (+)-JQ1. In the CCLE data set, expression of the target genes, *BRD2*, *BRD3* or *BRD4*, at naive state was confirmed in the S2-like cell lines, which are already known to exhibit MYC pathway activation (Figure 4a).^4^ As expected, the S2-like cell lines showed higher sensitivity to (+)-JQ1 as indicated by lower half maximal inhibitory concentration (IC_50_) values (Figure 4b), further supporting the use of hepatoma cell lines as experimental models to assess molecular subtype-specific anti-HCC drug response.

## Discussion

This study provides comprehensive reference information for a panel of widely used and commercially available hepatoma cell lines as *in vitro* models of human HCC molecular subtypes. Alternative strategies such as patient-derived xenograft (PDX) could enable personalized molecular evaluation for each patient.^19^ However, the low success rate and long time required to establish viable lines limit their flexible use. In contrast, despite the well-recognized limitations such as lacked tissue architecture and crosstalk with other cell types, cancer cell lines remain one of the major experimental models for anti-cancer drug discovery and assessment as a tractable system easily amenable to high-throughput and high-content molecular analysis.^20, 21^

Modeling of specific disease contexts such as clinical/molecular tumor subtypes is generally attempted by genetic engineering in experimental systems. However, it is practically infeasible to reconstitute the full spectrum of complex genetic and epigenetic molecular aberrations generally observed in non-hereditary solid cancers. Our study highlights an advantage of using existing hepatoma cell lines that inherently exhibit and recapitulate naturally occurring transcriptomic dysregulation observed in two of the three consensus human HCC molecular subtypes, which represent more advanced and aggressive tumors and therefore should be prioritized for therapeutic development. In fact, multiple preclinical studies and clinical trials support this concept. Dasatinib (Src/Abl inhibitor) was more effective in the S1-like cells.^6^ S2-like cells were more susceptible to epidermal growth factor receptor (EGFR) and insulin-like growth factor 1 receptor (IGF1R) inhibition.^22, 23^ BGJ398 (pan-fibroblast growth factor receptor inhibitor) elicited *in vivo* anti-tumor effect in xenograft of the S2-like cells.^8^ Epitope-optimized genetic vaccines targeting AFP (S2 marker)-induced specific CD8 T-cell-mediated killing of HCC cells in mice.^24^ Galunisertib, which targets the TGFβ pathway, a hallmark of the S1 subtype, is currently under evaluation in a phase 2 trial (ClinicalTrials.gov, NCT01246986).^25^ Tivantinib (MET inhibitor),^26^ ramucirumab (VEGFR2 inhibitor, showed better response in AFP-high patients)^9^ and GC33 (humanized monoclonal antibody against glypican 3 [GPC3])^27, 28^ were tested in clinical trials and could be more effective in the S2 subtype.

In our analysis of 25 hepatoma cell lines and one immortalized hepatocyte line, we could not find any cell line with the features of the S3 subtype. This may indicate that HCC cells in S3 tumors, clinically more differentiated and associated with better prognosis, are not capable of survival and proliferation in culture without support from the physiological liver tissue architecture. It is important to note that there is certain diversity in the characteristic molecular features for each subtype between the cell lines. Therefore, presence or absence of specific features (for example, AFP secretion status), which are potentially relevant to mechanism of action for agents to be tested, must be confirmed before picking cell lines for evaluation. In addition, differences in the sources and/or passages could alter the status of transcriptomic subtypes and other genetic aberrations, which ideally should be verified prior to the assessment. In summary, noting these limitations, hepatoma cell lines with designated HCC subtypes represent an invaluable resource as experimental models that retain the intra/inter-tumor/patient heterogeneity for anti-cancer drug assessment and development.

## Figures and Tables

**Table 1:** Characteristics of 25 hepatoma cell lines

*Cell line*	*Histological type*	*HBV*	*HCV*	*AFP*	*DCP*	*Race*	*Age*	*Sex*	*Source*	*ID*	*Reference*
Hep 3B2.1-7	HCC	(+)	NA	(+)	(+)	Black	8	M	ATCC	HB-8064	^29^
huH-1	HCC	(+)	NA	(+)	NA	Asian	53	M	JCRB	JCRB0199	^30^
Huh-7	HCC	(−)	NA	(+)	NA	Asian	57	M	RIKEN RCB, JCRB	RCB1366, JCRB0403	^30^
JHH-1	HCC	(−)	NA	(+)	NA	Asian	50	M	JCRB	JCRB1062	^31^
JHH-2	HCC	(−)	NA	(+)	NA	Asian	57	M	JCRB	JCRB1028	^31^
JHH-4	HCC	(−)	NA	(+)	NA	Asian	51	M	JCRB	JCRB0435	^32^
JHH-5	HCC	(−)	NA	(+)	NA	Asian	50	M	JCRB	JCRB1029	^31^
JHH-6	HCC	(−)	NA	(−)	NA	Asian	57	F	JCRB	JCRB1030	^31^
JHH-7	HCC	(+)	NA	(+)	NA	Asian	53	M	JCRB	JCRB1031	^33^
Li-7	HCC	(−)	(−)	(+)	NA	Asian	45	M	RIKEN RBC	RCB1941	---
PLC/PRF/5	HCC	(+)	NA	(+)	(+)	Black	24	M	ATCC, JCRB	CRL-8024, JCRB0406	^34^
SK-HEP-1	HCC	(−)	NA	(−)	(−)	White	52	M	ATCC	HTB-52	---
SNU-182	HCC	(+)	NA	(−)	NA	Asian	24	M	ATCC	CRL-2235	^35^
SNU-387	HCC	(+)	NA	(−)	NA	Asian	41	F	ATCC	CRL-2237	^35^
SNU-398	HCC	(+)	NA	(−)	NA	Asian	42	M	ATCC	CRL-2233	^35^
SNU-423	HCC	(+)	NA	(−)	NA	Asian	40	M	ATCC	CRL-2238	^35^
SNU-449	HCC	(+)	NA	(−)	NA	Asian	52	M	ATCC	CRL-2234	^35^
SNU-475	HCC	(+)	NA	(−)	NA	Asian	43	M	ATCC	CRL-2236	^35^
SNU-761	HCC	(+)	NA	(+)	NA	Asian	49	M	KCLB	KCLB00761	^36^
SNU-878	HCC	(+)	NA	(−)	NA	Asian	54	F	KCLB	KCLB00878	^36^
SNU-886	HCC	(+)	NA	(+)	NA	Asian	57	M	KCLB	KCLB00886	^36^
Hep G2	Hepatoblastoma	(−)	(−)	(+)	NA	White	15	M	ATCC, JCRB	HB-8065, JCRB1054	---
C3A	Hepatoblastoma	(−)	(−)	(+)	NA	White	15	M	ATCC	CRL-10741	---
HLE	Undifferentiated hepatoma	(−)	NA	(−)	NA	Unknown	68	M	JCRB	JCRB0404	^37^
HLF	Undifferentiated hepatoma	(−)	NA	(−)	NA	Unknown	68	M	JCRB	JCRB0405	^37^

Abbreviations: AFP, α-fetoprotein; DCP, des-gamma-carboxy prothrombin; ATCC, American Type Culture Collection (https://www.atcc.org); HBV, hepatitis B virus; HCC, hepatocellular carcinoma; HCV: hepatitis C virus; JCRB, Japanese Collection of Research Bioresources (http://cellbank.nibiohn.go.jp/english); KCLB, Korean Cell Line Bank (http://cellbank.snu.ac.kr/english); NA, not applicable; RIKEN BRC: RIKEN Bioresource Center (http://en.brc.riken.jp/index.shtml).
